# Hemophagocytic syndrome after living donor liver transplantation: a case report with a review of the literature

**DOI:** 10.1186/s40792-018-0505-5

**Published:** 2018-08-29

**Authors:** Norifumi Iseda, Tomoharu Yoshizumi, Takeo Toshima, Akinari Morinaga, Takahiro Tomiyama, Junichi Takahashi, Takashi Motomura, Yohei Mano, Shinji Itoh, Noboru Harada, Toru Ikegami, Yuji Soejima

**Affiliations:** Department of Surgery and Science, Graduate School of Medical Sciences, Kyushu University, 3-1-1 Maidashi, Higashi-ku, Fukuoka, 812-8582 Japan

**Keywords:** Hemophagocytic syndrome, Liver transplantation, Small-for-size syndrome

## Abstract

**Background:**

Hemophagocytic syndrome (HPS) is a rare and potentially fatal complication following liver transplantation.

**Case presentation:**

A 63-year-old woman with decompensated liver cirrhosis secondary to hepatitis B virus infection underwent living donor liver transplantation using the right posterior section of her husband’s liver (graft volume, 581 g; 56.8% of the recipient’s standard liver volume). She developed small-for-size syndrome on postoperative day (POD) 7, and HPS was diagnosed on POD 12 by bone marrow aspiration (white blood cells, 300/μL; neutrophils, 30/μL). Given that she tested negative for viral (hepatitis B virus and cytomegalovirus) and bacterial infections, it was considered likely to be secondary HPS. Steroid pulse therapy was initiated, and her white blood cell count increased to 4290/μL on POD 15, indicating that her peripheral blood leukocytes had improved. There were no surgical complications, but the patient died of prolonged graft dysfunction with bacterial sepsis on POD 14.

**Conclusions:**

We report a rare case of HPS occurring 2 weeks after living donor liver transplantation with a right posterior section graft, diagnosed early via bone marrow aspiration. This clinical course implies an association between HPS and graft dysfunction such as small-for-size syndrome. Further studies of the mechanism of hypercytokinemia-induced HPS are required to confirm the optimal treatment for HPS.

## Background

Hemophagocytic syndrome (HPS) is a rare but potentially fatal complication following liver transplantation [1]. HPS can be difficult to diagnose both clinically and histologically, with delays in diagnosis leading to significant morbidity and mortality. HPS can be either primary, with a genetic etiology, or secondary, associated with malignancies, autoimmune diseases, or infections such as tuberculosis or other viral, bacterial, fungal, or parasitic infections. The precise mechanisms have not been elucidated [2], though some studies have reported a relationship between HPS and small-for-size syndrome (SFSS) with graft dysfunction [1, 3].

We present the case of a patient who underwent living donor liver transplantation (LDLT) using a right posterior section graft who subsequently developed HPS and was treated with a combination of intravenous immunoglobulin, granulocyte-colony stimulating factor (G-CSF), calcineurin inhibitor conversion, and steroid pulse therapy.

## Case report

A 63-year-old woman with decompensated liver cirrhosis secondary to hepatitis B virus (HBV) infection was referred as a candidate for LDLT. She had been diagnosed with hepatitis B 20 years before, but it had not been actively treated. She had received best supportive care, but she and her family chose to proceed with LDLT. Laboratory findings before LDLT were as follows: serum total bilirubin, 8.4 mg/dL; serum albumin, 2.5 g/dL; prothrombin time, 40%; platelet count, 84,000/μL; and Model for End-stage Liver Disease score, 17. A large amount of ascites, liver atrophy, and collaterals were observed on computed tomography scan. At the time of admission, her urine volume was decreased to 50 mL/day, and continuous hemodiafiltration treatment was started for renal failure. The predictive risk score [4] was 0.80, which was lower than the score of 1.3 which predicts a poor prognosis, and the risk of postoperative mortality was therefore expected to be high. After obtaining full informed consent from both the donor and the recipient and approval from the Liver Transplantation Committee of Kyushu University, the patient was prepared for LDLT using a right posterior section graft.

The donor was the patient’s husband, who was 63 years old and had an identical blood type B. The surgical techniques were carried out as described previously [5]. The graft weight was 581 g, which was equivalent to 56.8% of the recipient’s standard liver volume (graft–recipient weight ratio, 1.12%). The hepatic arterial flow in the RHA was 87 mL/min, and the portal venous flow was 510 mL/min after reperfusion. The portal system pressure was 18 mmHg at the end of surgery, and splenectomy was not performed. The anhepatic time, and cold and warm ischemic times were 158 min, 92 min, and 49 min, respectively. The surgical time was 10 h, and the estimated blood loss was 4440 g. Ten units of red blood cells, 16 units of frozen plasma, and 40 units of platelets were transfused during surgery.

**Fig. 1:**
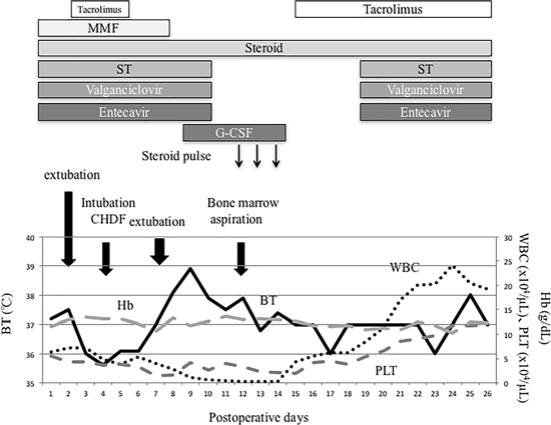
Clinical course after liver transplantation. WBC, white blood cell count; PLT, platelet count; BT, body temperature; G-CSF, granulocyte-colony stimulating factor; MMF, mycophenolate mofetil; ST, trimethoprim/sulfamethoxazole; Hb, hemoglobin

The post-transplant course is shown in Fig. 1. The patient received post-transplant immunosuppression with tacrolimus, steroid tapering, and mycophenolate mofetil. On postoperative day (POD) 7, the patient developed SFSS with serum total bilirubin 20.0 mg/dL and abdominal ascites 2000 mL/day [6], indicating prolonged cholestasis and intractable ascites [7]. Tests for HBV DNA and cytomegalovirus (CMV) were negative, and repeated blood cultures were also negative until POD 24. There were no signs of abnormal hepatic flow or surgical complications detected by daily Doppler echo until POD 14 and by routine computed tomography scan on POD 7. There was also no notable elevation of hepatobiliary enzymes, thus ruling out the possibility of graft rejection.

**Fig. 2:**
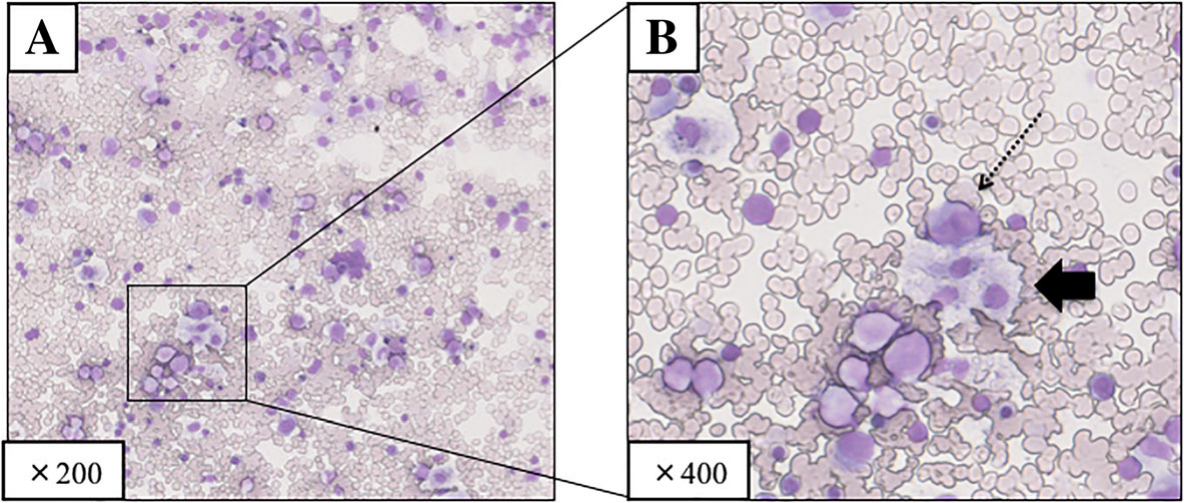
Bone marrow aspiration revealed many macrophages showing phagocytosis of hematopoietic cells. Dotted arrow shows macrophages phagocytosing neutrophils; thick arrow shows macrophages phagocytosing platelets. **a** Low-power view, × 200; **b** high-power view, × 400

The patient developed a fever above 39 °C on POD 9, and laboratory findings showed a white blood cell (WBC) count of 1120/μL and a platelet count of 40,000/μL, which fell to 300/μL (neutrophils, 30/μL) and 25,000/μL, respectively, on POD 11. She was treated with a combination of G-CSF and intravenous immunoglobulin, but her leukocytopenia/neutropenia failed to improve. Bone marrow aspiration was performed on POD 12, and histology revealed many macrophages and phagocytosis of hematopoietic cells (Fig. 2). The patient was therefore diagnosed with HPS following LDLT. Steroid pulse therapy with 1000 mg/day methylprednisolone was initiated on the same day and continued for 3 days. Her WBC count increased to 4290/μL on POD 15, suggesting an improvement in her peripheral blood leukocytes. However, the patient developed sepsis with a fever above 39 °C on POD 24, and blood cultures were positive for *Enterococcus faecium* and *Escherichia coli* infections. She recovered rapidly with empiric antibiotic therapy including carbapenem and vancomycin, but her graft dysfunction was prolonged with a consistent serum bilirubin value of around 30 mg/dL, and her renal failure never improved. She also had several episodes of bacterial pneumonia. Splenic artery embolism was performed on POD 84 but failed to improve her graft dysfunction. Regarding immunosuppressive treatment to control infections, mycophenolate mofetil was stopped on POD 8 and not restarted, and the minimum dose of tacrolimus was administered to maintain a trough blood concentration of 3–5 ng/mL. The patient underwent a tracheotomy, and her liver function was expected to improve with long-term management; however, she developed bacterial sepsis and died of liver failure on POD 146.

### Discussion

HPS, also referred to as hemophagocytic lymphohistiocytosis, is a rare and often fatal disease despite treatment [2], characterized by a variety of symptoms including fever, lymphadenopathy, hepatosplenomegaly, jaundice, and skin rash [8]. We report a rare case of a 63-year-old woman who developed HPS 2 weeks after LDLT with a right posterior section graft, who was treated with steroid pulse therapy following an early diagnosis via biopsy. This case may help to shed light on the relationship between HPS and graft dysfunction, including prolonged SFSS.

HPS is defined as a proliferation of phagocytic macrophages in the bone marrow, spleen, or lymph nodes, with clinical findings including fever for ≥ 7 days with peaks ≥ 38.5 °C, cytopenia (at least two of three lineages), and splenomegaly [9]. However, this definition is based on infant HPS and may be difficult to apply in adults and patients with various basic diseases such as liver cirrhosis or after LDLT. It is therefore necessary to perform bone marrow aspiration immediately to reach an early diagnosis of HPS in these patients [1]. The laboratory findings in the current patient showed WBC counts of 1120/μL on POD 9 and 300/μL on POD 11. Apart from HPS, other differential diagnoses such as medicine-induced disease, infections such as sepsis, hypersplenism, leukemia, and lack of vitamin B12 were unlikely and bone marrow aspiration on POD 12 showed typical HPS characteristics of phagocytic hematopoietic cells. This rapid diagnosis allowed early treatment with steroid pulse therapy, which contributed to an improvement in the patient’s peripheral blood leukocytes.

HPS is a rare complication after liver transplantation with a prognosis for patient survival of 50% [1, 8]. Fifteen patients, including the present case, have been reported to date (Table 1) [1, 3, 10–17], only four of whom survived. About a third of secondary HPS cases are associated with virus infection, half with lymphoma, and the remainder with bacterial and fungal infections. Given that HBV DNA and CMV tests on POD 7 were negative and blood cultures were also negative until POD 24, it was considered highly unlikely that the secondary HPS in the current patient was associated with a viral or bacterial infection. The combination of anemia and thrombopenia also added the possibly of thrombotic microangiopathy as a differential diagnosis, but no typical hemolytic anemia was detected in the bone marrow biopsy and there was no sign of encephalopathy. Thrombotic microangiopathy was thus discounted as a possible cause of HPS, and leukopenia was therefore considered the most likely cause. However, liver biopsy after LDLT and measurement of ADAMTS-13 were not performed in the present patient.

**Table 1:** Summary of 15 reports of hemophagocytic syndrome after liver transplantation

First author	No.	Age (years)	Sex	Donor type	Diagnosis	Onset	Causes	Treatment					Prognosis
						(POD)		G-CSF	IVIg	Steroid	Dialysis	Chemotherapy	
Chisuwa (8)	1	9 mo	F	Living	Biliary atresia	15	Unknown	–	+	+	PE	Etoposide	Died
	2	11 mo	M	Living	Biliary atresia	134	EBV	+	–	+	–	–	Died
Hardikar (13)	3	26 mo	M	Deceased	Acute liver failure	15	CMV	+	+	–	–	–	Alive
George (9)	4	10	M	Deceased	Acute liver failure	6 years	EBV	–	–	+	+	Etoposide	Alive
Taniai (12)	5	37	F	Living	Unknown	11	Unknown	–	+	+	PE, CHDF	–	Died
Karasu (10)	6	38	M	Living	HBV/HDV	124	Unknown	+	+	–	PE	–	Alive
Akamatsu (14)	7	48	F	Living	LC/HCV	50	HCV	+	–	–	–	–	Died
Dharancy (15)	8	49	F	Deceased	polycystic liver/kidney	12	HHV6	–	–	–	–	–	Died
Akamatsu (14)	9	49	F	Living	PBC	315	CMV	–	+	+	PE	–	Died
Soyama (3)	10	57	M	Living	LC/HCV/HCC	32	CMV/HCC	+	+	+	–	–	Died
Akamatsu (14)	11	59	M	Living	LC/HCV	138	Aspergillus	–	–	–	–	–	Died
Soyama (3)	12	63	M	Living	LC/HCV/HCC	81	Unknown	+	+	+	–	–	Died
Llado (11)	13	63	M	Deceased	Autoimmune hepatitis	NA	Unknown	–	+	+	–	–	Died
Yoshizumi (1)	14	63	M	Living	PBC	13	SFSS	+	+	+	–	–	Alive
Present case	15	63	F	Living	HBV	7	SFSS	+	+	+	CHDF	–	Died

*CHDF* continuous hemodiafiltration, *CMV* cytomegalovirus, *EBV* Epstein-Barr virus, *G-CSF* granulocyte-colony stimulating factor, *HBV* hepatitis B virus, *HCV* hepatitis C virus, *HCC* hepatocellular carcinoma, *HDV* hepatitis D virus, *HHV6* human herpesvirus 6, *IVIg* intravenous immunoglobulin, *LC* liver cirrhosis, *mo* months, *NA* not available, *PBC* primary biliary cholangitis, *PE* plasma exchange, *POD* postoperative day, *SFSS* small-for-size syndrome

In general, patients may develop secondary HPS via hypercytokinemia [18] due to systemic inflammatory response syndrome associated with a viral or bacterial infection. The current LDLT recipient had SFSS, which might have been associated with the donor’s older age (63 years) [19]. SFSS can also induce oxidative stress and hypercytokinemia [20].

Based on this hypercytokinemia theory, 10 of the past 15 cases (66.7%) were treated with steroids, 10 (66.7%) with intravenous immunoglobulin, and four (26.7%) with plasma exchange. The present patient started combination therapy with intravenous immunoglobulin, G-CSF, calcineurin inhibitor conversion, and steroid pulse therapy on POD 12, with subsequent improvement in her peripheral blood leukemia. However, her course was complicated by bacterial sepsis on POD 24. The causal relationship between steroid pulse therapy and sepsis could not be clarified, but the possibility of a septic attack is always present.

In addition, the relationship between SFSS and HPS was not unknown in the present case, and the possible causative effect of SPSS remains speculative. To reduce the risk of HPS, it is necessary to avoid developing viral and bacterial infections and hypercytokinemia, which induce secondary HPS, while the use of elderly donors might also increase the risk of SFSS. However, it may not be possible to avoid these factors in the setting of LDLT. Further studies on the mechanism of hypercytokinemia-induced HPS and more basic studies are needed to confirm the optimal treatment for HPS.

## Conclusions

We report a rare case of HPS that occurred 2 weeks after LDLT using a right posterior section graft, with an early diagnosis made via bone marrow aspiration. The patient died of liver failure with accompanying bacterial sepsis, but the disease course implied an association between HPS and graft dysfunction, such as SFSS.

## Abbreviations

G-CSF: Granulocyte-colony stimulating factor
HBV: Hepatitis B virus
HPS: Hemophagocytic syndrome
LDLT: Living donor liver transplantation
POD: Postoperative day
SFSS: Small-for-size syndrome
WBC: White blood cell
